# Acupuncture Alleviated the Nonmotor Symptoms of Parkinson's Disease including Pain, Depression, and Autonomic Symptoms

**DOI:** 10.1155/2014/953109

**Published:** 2014-12-31

**Authors:** Chifumi Iseki, Taiga Furuta, Masao Suzuki, Shingo Koyama, Keiji Suzuki, Tomoko Suzuki, Akiyo Kaneko, Tadamichi Mitsuma

**Affiliations:** ^1^Department of Kampo Medicine, Aizu Medical Center, Fukushima Medical University, 21-2 Maeda, Tanisawa, Kawahigashi, Aizuwakamatsu, Fukushima 969-3492, Japan; ^2^Department of Neurology, Hematology, Metabolism, Endocrinology and Diabetology, Yamagata University Faculty of Medicine, 2-2-2 Iida-Nishi, Yamagata 990-2331, Japan; ^3^Department of General Internal Medicine, Aizu Medical Center, Fukushima Medical University, 21-2 Maeda, Tanisawa, Kawahigashi, Aizuwakamatsu, Fukushima 969-3492, Japan

## Abstract

A woman started to feel intractable pain on her lower legs when she was 76. At the age of 78, she was diagnosed as having Parkinson's disease (PD). The leg pain was suspected to be a symptom of PD after eliminating other causes. The patient also suffered from nonmotor symptoms, depression, anxiety, hot flashes, and paroxysmal sweating. Though the patient had received pharmacotherapy including levodopa for 5 years, she still suffered from the nonmotor symptoms and was referred to our department. We treated her with acupuncture based on the Chinese traditional medicine and electroacupuncture five times per week. After the 2-week treatment, the assessment for the symptoms was as follows; visual analogue scale (VAS) score of the leg pain was 16 mm (70 mm, before), Hamilton's rating scales for depression (HAM-D) score was 9 (18, before), timed 3 m Up and Go took 20 steps in 30 sec (24 steps in 38 sec, before), and the Movement Disorder Society-sponsored revision of the Unified Parkinson's Disease Rating Scale (MDS-UPDRS) Part 1 score was 13 (21, before). Autonomic symptoms, hot flashes and paroxysmal sweating, were also alleviated. Acupuncture may be a good treatment modality for nonmotor symptoms in PD.

## 1. Introduction

Nonmotor symptoms of Parkinson's disease (PD), for example, primary pain, psychiatric symptoms, and autonomic symptoms, are common but difficult to treat. We applied acupuncture to these symptoms of patient with PD.

## 2. Case Presentation

An 81-year-old woman with history of hypertension and lumber fracture without after effects was referred to our department complaining of right-dominant lower leg pain. The pain started when she was 76. She had visited several orthopedists and psychiatrists who prescribed nonsteroid anti-inflammatory drugs, etizolam and paroxetine, although the pain had remained and the cause of the pain also remained unclear. At the age of 78, a neurologist diagnosed her with Parkinson's disease (PD) due to her mask-like face and bradykinesia. Warmth, touch, and pain sensations were within normal limits, and the result of a nerve conduction study was also normal. Around that time, the patient began to suffer from hot flashes and paroxysmal sweating emerging in no regularity; the frequency ranged from several times per day to several times per week and had no association with symptoms of PD or the timing of levodopa administration. Although orthostatic hypotension was revealed by a Schellong test, she had never experienced orthostatic syncope until that time or thereafter. The patient was prescribed levodopa, which resulted in the mitigation of the lower leg pain, in addition to a kinetic effect. She was always dominated by anxious or depressive moods and complained about the lower leg pain, hot flashes, and paroxysmal sweating. The anxiety was partially alleviated after the prescription of duloxetine. At the age of 81, she was admitted to our department for therapy.

The patient's height was 141 cm and her body weight was 57 kg. Blood pressure was 132/72 mmHg and pulse was 81/min, a regular rhythm. She was experiencing hot flashes and paroxysmal sweating several times per day. Neurological examination showed that cranial nerves, muscle power, and deep tendon reflex were normal. Lasègue phenomenon was not induced. She expressed a slight tremor, a mask-like face, a low voice, bradykinesia, a forward-bent posture, and right-dominant mild rigidity in the extremities. Dystonia was not observed. The pain emerged in approximately half of her lower leg where she felt a “tingling” at rest, with no worsening when walking. The pain occurred without a disturbance of sensation, including hyperesthesia, and was not distributed into some dermatomes. Leg pain was valued by Visual Analogue Scale (VAS) to be 70 mm on the right and 54 mm on the left. However, the strength of pain was changeable throughout the day and was also not associated with other symptoms or the administration of levodopa. She was not diagnosed as having restless legs syndrome. The patient was in a constant state of anxiety about every symptom. Her Hamilton depression scale (HAM-D) score was 18, but she had no suicidal or guilty thoughts. The lumbar MRI showed spinal canal stenosis in L1/2 and L4/5; however, intervertebral foramina were open. The Movement Disorder Society-sponsored revision of the Unified Parkinson's Disease Rating Scale (MDS-UPDRS) score was as follows: Part 1 (nonmotor aspects of experiences of daily living): 21, Part 2 (motor experiences of daily living): 12, and Part 3 (motor examination): 26. The timed 3 m Up and Go test result was 24 steps in 38 seconds. The scores were all assessed at approximately 3 hours after the administration of levodopa. This was considered to be “on” time, though the patient had not experienced a significant “off” time.

**Figure 1:**
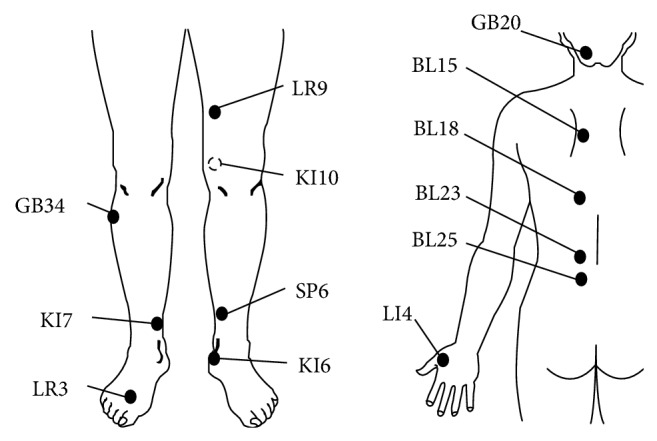


She had continued to take 500 mg/day of levodopa, 300 mg of entacapone, 1.5 mg of pramipexole, and 20 mg of duloxetine. We applied acupuncture treatment to the nonmotor symptoms five days per week, using the LR3, LI4, KI5, KI7, SP6, GB34, BL18, BL15, and GB20 acupuncture points, with needles placed for 10 minutes. We also applied electroacupuncture (1 Hz, 7 minutes with Ohm Pulser LFP-400A) on the KI10, LR9, BL23, and BL25 points, for the purpose of loosening the muscle tension (Figure 1). The reasons for selecting the particular acupuncture points (acupoints) for the present case were as follows: the acupoints known as LR3, KI6, and KI7 on the lower leg were expected to relieve pain. The acupoints for depression and autonomic symptoms were selected according to the theory of traditional Chinese medicine; the acupoints known as BL15, LR3, and SP6 were for depression and anxiety, and LR3, KI7, BL18, BL15, and GB34 were for hot flashes and paroxysmal sweating. We used disposable needle of 40–50 mm length, 0.14–0.18 mm diameter (made by SEIRIN Co. Ltd). To prevent disuse, we prescribed the physical therapy, muscle training and walking practice five times per week.

Immediately after every acupuncture treatment session, she felt that her legs had become light. After two weeks, her lower leg pain was milder, indicated by the 10.5 mm VAS score on both sides. The daily attacks of hot flashes and paroxysmal sweating were decreased to about three times per week. Depression and anxiety were obviously lessened, which resulted in the HAM-D score of 9 and also in the elimination of the MDS-UPDRS Part 1 score of 13. The scores of Part 2 and Part 3 were 10 and 23, respectively. Her steps became larger and bradykinesia was weakened; therefore, in the timed 3 m Up and Go test, she was able to walk 20 steps in 30 seconds.  Table 1 shows the pre- and posttreatment assessment summary.

After the 3 weeks' acupuncture therapy, she discharged and continued to receive monthly acupuncture therapy in the outpatient department.

## 3. Discussion

We treated the patient of PD with acupuncture, which alleviated the nonmotor symptoms of PD, the lower leg pain and psychiatric and autonomic symptoms.

The lower leg pain, which was the patient's primary complaint, was not accompanied by typical features of neuropathy, radiculopathy, or dystonia. However, it was difficult to completely deny the existence of such pathology. Although we should leave the possibility that her chronic pain was caused by a complex of some type, we suppose that her pain was associated with PD. Pain is common nonmotor symptoms in PD patients, as studied by O'Sullivan et al., who reported that 21% of the patients with PD had complicated nonmotor symptoms and 15% had complicated pain [1]. Pain in PD can precede the diagnosis of the disease [2], as the pain in the present case did. There is no consensus for the medication of pain in PD [2]. The present case had a history of the lower leg pain alleviated after the levodopa therapy started. Possible PD pathology associated with the patient's pain was weakened by the levodopa. PD is a multifocal degenerative and progressive disease. Therefore it could affect the pain process at multiple levels, from the transmission of pain from peripheral structures to the higher centers, to its perception and interpretation [2]. For example, the denervation of nigrostriatal system in PD changes the neuronal activities involving the lateral thalamus, which plays an important role for central pain [2]. In patients with PD, the chemical messengers modulating the pain, 5-hydroxy-indole acetic acid, *β*-dndorphin, and met-enkephalin, are also found to have decreased CSF level [3]. Acupuncture seems to be a clinical application for any kind of pain. However, the mechanism of its analgesic effect is not completely understood. Acupuncture may alter the metabolism of substrates involved in both the ascending pathways and the descending inhibitory pain pathways [4]. Acupuncture triggers the release of encephalin and endorphin in the periaqueductal gray (PAG) and so forth [5]. By increasing those endogenous opioids and by complementing the degenerated pain processing in PD, acupuncture may also be effective for the pain of PD.

Depression directly related to the underlying pathophysiology of PD is characterized as the following: absence of history of depression, absence of guilty thoughts and self-blame, absence of suicidal behavior, and right-sided onset [6]. The present case fulfilled those characteristics. Psychiatric symptoms including depression and anxiety are also clinically important and are associated with quality of life. The pathophysiology of these psychiatric symptoms reflects the complexity of involvement of neurotransmitters, including dopaminergic, serotonergic, noradrenergic, and cholinergic system [7]. It was reported that serotonin reuptake inhibitors (SSRI) or serotonin and noradrenalin reuptake inhibitors (SNRI) improved these psychiatric symptoms in PD [8]. In the present case, the anxiety was partially improved by SNRI; however, the patient's mood disorder fluctuated and continued. A recent clinical trial showed that acupuncture plus usual medical care reduced the depression more effectively, compared with usual medical care alone [9]. Her mood might have been influenced by the pain relief. Pain and mood are difficult to discuss independently. From observing this case, we are able to mention that acupuncture was effective for depression in PD, possibly as a result of both direct and indirect action for pain alleviation. Before starting acupuncture, an SNRI known as duloxetine had been prescribed for three years as a basal mood treatment. In addition to this basal treatment and acupuncture, physical therapy might also have had an effect on the patient's state of depression and anxiety. Numerous studies have revealed that physical exercise can ease depression in the elderly, by changing both the physiology and psychology of the subjects [10]. In the present case, these multiple effects promoted the recovery of the patient's mood. We should recognize that acupuncture is one of many therapeutic approaches that can be used to treat mood disorders.

The hot flashes and paroxysmal sweating that the patient had suffered from for a long time were reduced by acupuncture. Autonomic symptoms are common in PD, as described in one study as a complication in up to 50% of patients [10]. Sweat disturbances occur in 30–50% of PD patients [11]. These symptoms affect the quality of life and state of mind of patients with PD [11]. However, effective therapies are rare, especially for sweat disturbances and hot flash. Vasomotor symptoms associated with menopause have been proven to be decreased by acupuncture compared with placebos [12]. The frequency of hot flashes was reduced by over 50% when acupuncture was administered once a week for 12 weeks [12]. There are few studies of acupuncture that focus on sweating. Clinically, menopausal hot flashes often emerge with sweating; therefore it is supposed that acupuncture also treat sweat disturbances. The psychiatric effect of acupuncture also may have a role in reducing autonomic symptoms, and a relaxation therapy program has reduced hot flashes of menopausal symptoms [12]. Acupuncturists commonly treat general conditions of any kind of disease [13] including the aforementioned psychiatric symptoms and autonomic symptoms, which are common but naturally difficult to treat. However, most medical providers are not aware of this. Acupuncture may provide an effective treatment for autonomic symptoms.

Motor functions of the present case expressing in 3 m Up and Go task were also improved after the treatment. We suppose that the combination of acupuncture and physical therapy affected this. A study by Shulman et al., who treated 20 patients with PD, demonstrated the difficulty of showing the effect on motor symptoms, validated with UPDRS, and so forth. However, 85% of patients reported the subjective improvement of motor and nonmotor symptoms [14].

Acupuncture rarely causes adverse effects and is well tolerated in patients with PD [14], which was also found in our case. It is possible that acupuncture affects both nonmotor and motor symptoms of PD. However, with only the results of this case, we are not able to come to a solid conclusion regarding the effectiveness of acupuncture. A controlled trial of a therapy for PD found the effect of the therapy in MDS-UPDRS score changes of about -3 points in part 1, and -5 points in Part 2 [15]. Referencing these change of the MDS-UPDRS score, we considered that our treatment improved various symptoms of the patient's PD expressed in the MDS-UPDRS score. Acupuncture may be a good choice of treatment, especially for patients suffering from nonmotor symptoms of PD where there is a lack of treatment. We are only able to report a single case of PD here. However, we suppose that acupuncture therapy is worth applying to other PD patients especially expecting the effects on nonmotor symptoms. Future clinical studies should be undertaken with a larger number of PD patients in order to further investigate this matter.

## Figures and Tables

**Table 1:** The assessment before and after two-week treatment of acupuncture.

	Before	After
Lower leg's pain: VAS (right/left)	70/45	16/5
MDS-UPDRS		
Total	**59**	**46**
Part 1	21	13
Part 2	12	10
Part 3	26	23
Timed 3 m Up and Go	38 sec, 24 steps	30 sec, 20 steps
Hamilton's depression scale (HAM-D)	18	9

Note: the Movement Disorder Society-sponsored revision of the Unified Parkinson's Disease Rating Scale, MDS-UPDRS.
